# The Predictive Value of A, B, and C-Type Natriuretic Peptides in People at Risk of Heart Disease: Protocol for a Longitudinal Observational Study

**DOI:** 10.2196/37011

**Published:** 2023-01-11

**Authors:** Timothy C R Prickett, John F Pearson, Richard W Troughton, Martin A Kennedy, Eric A Espiner

**Affiliations:** 1 Christchurch Heart Institute Department of Medicine University of Otago Christcurch New Zealand; 2 Department of Pathology and Biomedical Science University of Otago Christchurch New Zealand; 3 Biostatistics and Computational Biology Unit University of Otago Christchurch New Zealand

**Keywords:** Cardiovascular risk, arterial and LV elastance, polygenic risk scores, cGMP, bioactive and amino-terminal natriuretic peptide, heart disease, cardiovascular, middle age, risk, prediction, biomarkers, detection

## Abstract

**Background:**

Heart disease and stroke are major and often unheralded causes of serious morbidity and premature death in middle age. Early detection of those most at risk is an urgent unmet need for instituting preventative measures. In an earlier community study (Canterbury Health, Ageing and Life Course [CHALICE]) of healthy people aged 50 years, contrary to previous reports, low levels of the heart hormone B-type natriuretic peptide (BNP) were associated with reduced measures of heart function and higher markers of vascular risk. A specific gene variant (rs198358) was found to be an independent contributor to higher BNP levels. A closely related vascular hormone (C-type natriuretic peptide [CNP]) showed opposite associations—higher levels were correlated with higher vascular risk and reduced cardiac function. To determine whether these novel findings predict serious heart or vascular disease in later life, this proposal re-examines the same CHALICE participants 15 years later.

**Objective:**

The primary objective is to determine the predictive value of (1) low plasma concentrations of the circulating cardiac hormones (atrial natriuretic peptide [ANP] and BNP) and (2) high levels of the vascular hormone CNP at age 50 years in detecting impaired cardiac and vascular function 15 years later. Secondary objectives are to determine specific associations of individual analytes (ANP, BNP, CNP, cyclic guanosine monophosphate [cGMP]) with echo-derived changes in cardiac performance at ages 50 years and 65 years.

**Methods:**

All of the 348 participants (205/348, 58.9% female; 53/348, 15.2% Māori or Pacifica ethnicity) participating in the original CHALICE study—free of history of heart or renal disease at age 50 years and who consented to further study—will be contacted, recruited, and restudied as previously described. Data will include intervening health history, physical examination, heart function (speckle-tracking echocardiography), vascular status (carotid intimal thickness), and genetic status (genome-wide genotyping). Laboratory measures will include fasting blood sampling and routine biochemistry, ANP, BNP, CNP, their downstream effector (cGMP), and their bio-inactive products. Humoral metabolic-cardiovascular risk factors will be measured after an overnight fast. Primary outcomes will be analyzed using multiple linear regression.

**Results:**

The study will commence in 2022 and be completed in 2024.

**Conclusions:**

Proving our hypothesis—that low BNP and high CNP at any age in healthy people predict premature aging of heart and blood vessels, respectively—opens the way to early detection and improved outcomes for those most at risk. Confirmation of our hypotheses would improve current methods of screening and, in appropriate cases, enable interventions aimed at increasing natriuretic hormones and reducing risk of serious cardiovascular complications using drugs already available. Such advances in detection, and from interventional corrections, have the potential to not only improve health in the community but also reduce the high costs inevitably associated with heart failure.

**International Registered Report Identifier (IRRID):**

PRR1-10.2196/37011

## Introduction

**Figure 1:**
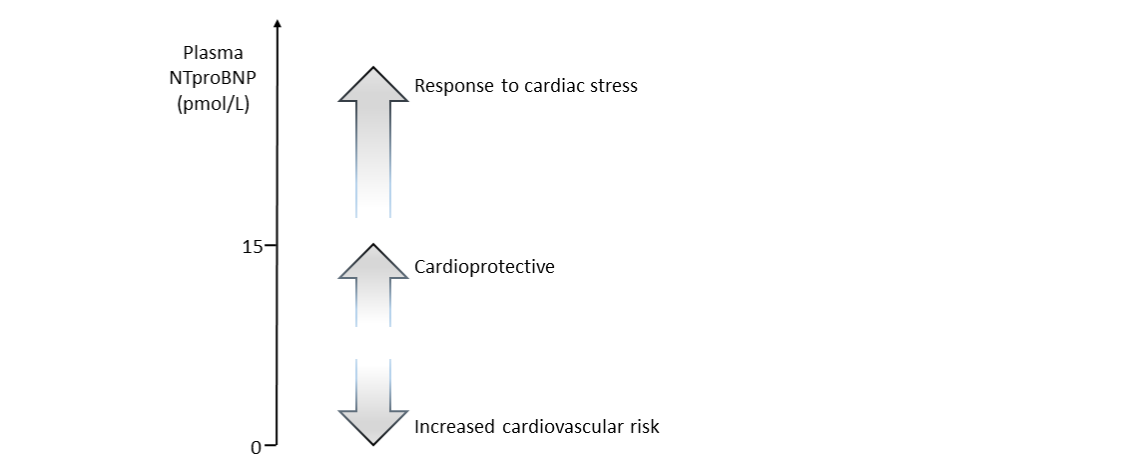
Beneficial and pathological circulating levels of aminoterminal B-type natriuretic peptide (NTproBNP) reflecting expression of the cardioprotective hormone BNP.

Atrial natriuretic peptide (ANP) and B-type natriuretic peptide (BNP) are cardiac peptides secreted by atrial and ventricular myocytes, respectively [1]. Both hormones circulate in plasma and, acting via a common guanylate receptor (NPR 1), serve to regulate the renal excretion of salt, lower systemic arterial pressure, and reduce intracardiac pressure [2]. A third member of the family (C-type natriuretic peptide [CNP]) acts in cardiac [3] and vascular intimal tissues [4] via a different guanylate cyclase receptor (NPR 2) to reduce inflammation and cell proliferation [4,5] and increase flow and pressure in the microcirculation [6]. In contrast to the endocrine and paracrine actions of ANP and BNP, actions of CNP are largely paracrine and, at levels found in healthy adults, are barely detectable in plasma. The bioactive forms (ANP, BNP, and CNP) of all 3 peptides have strong affinity for a third (non-guanylate cyclase) receptor (NPR 3) that internalizes and degrades these peptides. However, aminoterminal bio-inactive products of the prohormones synthetized in tissues (ie, aminoterminal ANP [NTproANP], aminoterminal BNP [NTproBNP], and aminoterminal CNP [NTproCNP]) are not NPR 3 ligands, have longer half lives in plasma, and serve as surrogates for the respective prohormone production in tissues [7-9]. Increase in intracardiac pressure is a strong stimulus to the secretion of the cardiac hormones, ANP and BNP, which are raised in patients with overt heart failure [2]. Factors regulating CNP are less clear but include cytokines [10] and shear stress [11] in vascular tissues. Numerous studies of community dwellers have shown that higher plasma concentrations of BNP products (BNP or NTproBNP) not only detect cardiac impairment but also predict adverse outcomes [12-14] in participants without evidence of heart disease. However, we found that, in participants without history of heart disease and with plasma concentrations of ANP and BNP within the normal range, higher values associate with lower markers of vascular and metabolic risk and with improved cardiac performance [15,16] (Figure 1). A specific gene variant (rs198358)—previously reported in a genome-wide association study (GWAS) to associate with lower blood pressure [17]—was found to be an independent contributor to BNP levels in 2 quite separate populations: one 28 years old [16] and another 50 years old [15]. Together with recent reports of other closely related gene variants in the community positively affecting BNP levels and reducing adverse cardiac outcome [18], we suggest that genetic factors drive the ANP and BNP links we identified with markers of cardiometabolic health. Acquired factors such as increased adiposity is associated with lower plasma concentrations of BNP [19]—possibly related to an effect of glycosylation on the assay of BNP in plasma [20]. Others have therefore suggested the recognized increase in adverse events such as the metabolic syndrome in obesity are consequent on a BNP-deficient state. Importantly, in our 2 previous community studies, lower BNP levels (which associate with markers of impaired metabolic health) were independent of adiposity.

**Figure 2:**
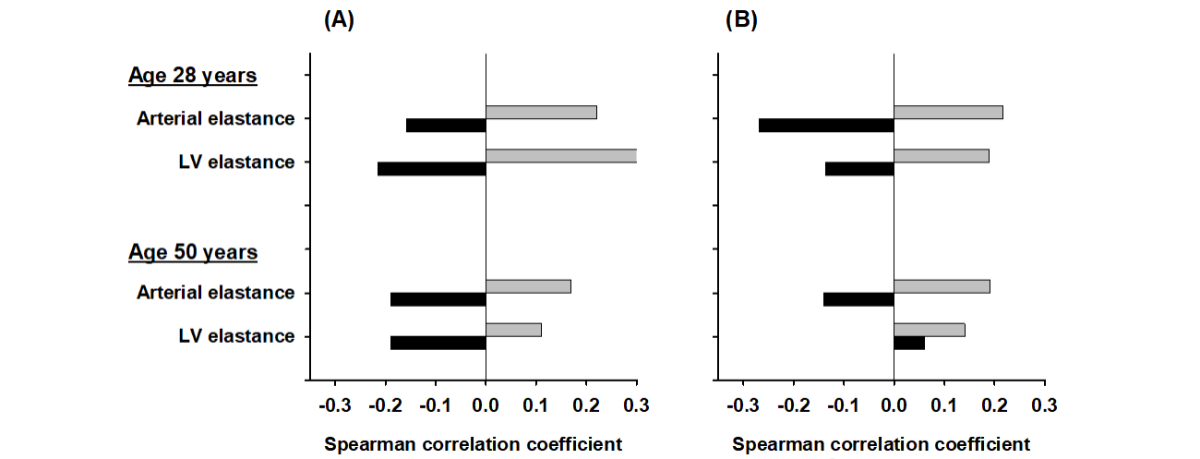
Comparison of Spearman correlation coefficients of aminoterminal B-type natriuretic peptide (NTproBNP; black bars) and aminoterminal C-type natriuretic peptide (NTproCNP; grey bars) with left ventricle (LV) and arterial elastance in healthy (A) men and (B) women aged 28 years and 50 years without a history of heart disease. Stiffer tissues equate with higher elastance. Coefficients >0.18 were significant.

Few, if any, studies have examined plasma concentrations of CNP products (CNP or NTproCNP) in this context. However, in both community studies [15,16], we found plasma CNP products—opposite to ANP and BNP—are higher in those with impaired cardiometabolic health. We suggest that these associations with CNP likely reflect an adaptive compensatory response to inflammation in vascular [21,22] and myocardial tissues [2,23,24]. Notably, in both age groups, plasma CNP was positively associated with markers of arterial and left ventricular stiffness in both sexes, whereas these associations with BNP were inverse except in older women (Figure 2).

The key question regarding the significance of these findings in predicting adverse cardiac or vascular outcome can only be answered by appropriate re-examination of the same participants at a later date.

Accordingly, here, we outline a proposal to comprehensively restudy participants aged 49 years to 51 years in the Canterbury Health, Ageing and Life Course study (CHALICE) study [15] undertaken 15 years previously. We postulate that (1) participants with lower levels of ANP or BNP at age 50 years will exhibit more advanced cardiac and metabolic impairment 15 years later and (2) higher levels of CNP products at age 50 years will associate with more advanced vascular and myocardial degenerative disease at age 65 years. Although protocols for the study of the predictive value of data on cardiovascular outcomes have been reported in specific disease states such as heart failure [25] or psoriasis [26], no previous study examining the predictive value of natriuretic peptides on cardiovascular outcomes in participants without evidence of heart disease has been reported to date.

## Methods

### Participants

All of the surviving 348 participants (205/348, 58.9% female; 53/348, 15.2% Māori or Pasifika ethnicity) participating in the CHALICE study [27] (free of history of heart or renal disease at age 50 years and who consented to further study) will be contacted, recruited, and restudied exactly as previously described [15]. Every effort will be made to re-establish contact with the CHALICE participants, including email, phone, surface mail, and cross checking with the electoral role, when necessary. Participants with cardiovascular events (ischemic heart disease, stroke, heart failure, atrial fibrillation) or renal disease developing since the previous study will be separately studied with respect to natriuretic peptide measurements at age 50 years but excluded in the main analysis of results at age 65 years. Records of hospital visits and admissions or death of participants since the previous review will be obtained from routine national data sets.

### Ethics and Governance

All current participants gave written consent, and ethical approval was given by the Southern Health and Disability Ethics Committee (reference URA/10/03/021). Written informed consent will be sought from all participants agreeing to participate in further study. Relationships with Māori will be strengthened, and the studies’ governance and policies will be updated to better align with Māori tikanga (customs) and to enhance Māori health outcomes [28].

### Data Collection

After obtaining informed written consent, the following data will be obtained: health history (chronic morbidities and medications, tobacco consumption); physical examination (including blood pressure, height, weight, BMI); heart function as measured using echocardiography including speckle-tracking strain [29]; carotid artery intimal thickness as measured during echo examinations; laboratory measures (including fasting urine and blood sampling for routine biochemistry and plasma storage for measurements of natriuretic peptides, cyclic guanosine monophosphate [cGMP], and other analytes). Previously established metabolic-cardiovascular risk factors will be measured after an overnight fast [15]. Plasma ANP, BNP, CNP (measured after extraction by radioimmunoassay), NTproANP, NTproBNP, NTproCNP, and cGMP will be assayed as previously described [16,30,31]. Because of links between CNP and bone turnover markers [32], plasma type 1 collagen C-telopeptide (CTx) will be measured in these older participants.

In order to generate data for genetic studies, including calculation of polygenic risk scores, DNA extracted from peripheral blood of all consenting participants will be genotyped on an appropriate platform such as the Infinium Global Screening Array (Illumina, San Diego, CA), and imputation will be carried out using standard approaches [33]. Polygenic risk scores will be generated based on existing data for large GWAS of relevant cardiac phenotypes [34].

The predictive value of natriuretic peptides at age 50 years for impaired atrial and ventricular function and for carotid intimal thickness at age 65 years will be determined using multiple variable analysis. In participants excluded from the main analysis because of interval cardiac or vascular disorder after the original study, natriuretic peptides at baseline and at 65 years will be studied in relation to cardiac and metabolic indices documented in the unaffected population and to type of event.

### Specific Objectives and Hypotheses Affecting ANP and BNP

Here, we hypothesize that (1) low BNP at 50 years old will be associated with impaired atrial and ventricular structure (volumes and mass) and function (atrial strain and ventricular ejection fraction) at 65 years compared with those with high BNP at baseline; (2) at age 65 years, the percentage increase of BNP in participants with low BNP at 50 years old will be significantly greater than in those with higher BNP at baseline; and (3) in women, for whom the inverse association of ANP/BNP with left ventricle (LV) elastance is lost at age 50 years (Figure 2), decline in cardiac performance at age 65 years will be greater than in men.

### Objectives and Hypotheses Affecting CNP

For the first time in humans, CNP has been linked to measures of arterial and LV stiffening at both ages and in both sexes (Figure 2). Here, we hypothesize that (1) plasma NTproCNP at age 50 years will predict decline in atrial and ventricular function as measured by speckle echocardiography at age 65 years, (2) plasma CNP products at age 50 years will predict increased atrial and ventricular stiffness and carotid intimal thickening at age 65 years, and (3) change in plasma concentration of CNP products 15 years later will be linked to markers of impaired cardiac performance and metabolic health at 65 years of age. Again, the contribution of a longitudinal study, undertaken at a period of increasing incidence of heart disease, will be crucial to providing proof of the independent association of CNP’s role in maintaining cardiovascular and metabolic health. Showing that CNP levels at 50 years of age heralds later risk of stroke or heart failure identifies likely preventive measures, including treatments augmenting CNP and BNP levels in blood.

### Other Objectives

Associations of polygenic risk scores with phenotype (measures of cardiac, metabolic, and vascular status) and associations with plasma natriuretic peptides will be studied. On the basis of previous findings from a single variant, we hypothesize positive links will be strengthened using polygenic risk scores.

Relevance of cGMP in the assessment of cardiac and vascular risk is an important new objective considering its role in mediating actions of all 3 natriuretic peptides. Based on prior work [35-37], we postulate that ANP will be independently associated with cGMP at age 50 years and age 65 years. Increasing recognition of CNP’s role in combatting myocardial stiffening and the higher sensitivity of the cGMP response to stimulation of the CNP receptor (natriuretic peptide receptor 2 [NPR2]) in cardiac tissues, compared with that of natriuretic peptide receptor 1 (NPR1), suggests that CNP will independently predict cGMP in women and strengthen further at 65 years of age.

### Statistics

The primary outcomes (hypotheses 1, 2, and 3) will be analyzed using multiple linear regression. Allowing for 30% nonresponse (n=244) and 10 covariates, this will be able to detect an f^2^ of 0.083 (small-to-medium Cohen effect size) with 80% power and α=.01, which includes a multiple testing correction equivalent to a Bonferroni correction for 5 independent predictors. Nonlinear relationships will be examined with generalized additive models, and all models will be graphically assessed for goodness of fit. Indices will be analyzed as continuous variables or categorized as appropriate. Additionally, mediation analysis [38] will be used to quantify the role of cGMP in the relationship between natriuretic peptides and cardiac structure and performance in the primary outcomes.

## Results

In the 10-year follow-up period after the initial study visit, 6 participants have died (none from cardiovascular causes), and 36 (10% of the cohort) were hospitalized for cardiovascular events. Ischemic heart disease (10 participants) was the most common reason for cardiovascular admission. Baseline NTproBNP or NTproCNP concentrations for men or women subsequently hospitalized for cardiovascular events were not significantly different from values of study participants remaining free from cardiovascular events.

The project and protocol have been approved by the National Ethics Committee. Having received funding from the New Zealand Lottery Board (Health), re-enrolment of CHALICE participants commenced in October 2022. At the date of this manuscript submission, 21 participants had successfully completed the protocol without incident.

## Discussion

Two separate community studies of participants free of heart disease showed that lower plasma concentrations of the cardiac hormones, ANP and BNP, are associated with increase in vascular stiffness and reduced metabolic health, whereas the opposite is true of CNP concentrations—lower levels associate with improved cardiometabolic health. These reproducible findings are independent of age and obesity. Restudy 15 years later of those aged 50 years will establish the value of natriuretic peptide levels in predicting adverse cardiac and vascular outcomes using clearly defined hypotheses based on data measured by the same laboratory using best practice quality control.

### ANP and BNP as Paracrine and Endocrine Regulators of Cardiovascular Status

In light of strong evidence in experimental animals of protean beneficial actions of ANP/BNP across many tissues affecting the integrity of the cardiovascular system [2,39] and the deleterious impact of ablating ANP/BNP pathway activity [40,41], it is unsurprising that circulating concentrations of ANP/BNP products within the normal range have beneficial effects in maintaining cardiovascular and metabolic health in healthy people [42,43]. Whether these subtle differences in plasma concentrations predict subsequent cardiovascular health is the crucial question addressed in this prospective study. That genetic factors contribute is evident from several studies identifying a single [15,16,44] or more variants [39,45-47] in *NPPA* or *NPPB*. Plausibly, the accumulative impact of these explain, in part, the significant impact of subtle increases in circulating levels on “ideal cardiovascular health” [48] in either sex if maintained from early life. Obtaining array-based genotypes in CHALICE participants, as now proposed, will allow us to more closely examine impacts of additional natriuretic peptide and receptor genetic variants over and above that (rs198358) identified previously. The array data will also enable calculation of polygenic risk scores for various cardiac phenotypes and disease states and allow evaluation of the predictive or prognostic value of these in relation to existing clinical measures and risk factors. The value of this approach to identifying participants most at risk has been emphasized in a recent review of atherosclerosis [49]. Acquired factors such as obesity—lowering BNP [19]—also need consideration and will be assessed by changes in BMI. Although both ANP and BNP activate the same receptor (NPR1), their variable expression, location, and metabolism highlight the need to measure both bioactive forms in plasma [41] in associative studies linking levels with changing indicators of vascular, metabolic, and cardiac function in the 15 years after middle age when the incidence of heart disease increases greatly. No other study has examined these relationships in healthy participants. The conditions and blood sampling for measurements proposed in the re-examination will be undertaken exactly as they were previously (overnight morning fasting in the rested seated position) at one central location (University of Otago Nicholls Clinical Research Centre) utilizing standardized assays of natriuretic peptides developed by research staff of the Christchurch Heart Institute. Importantly, CNP concentrations in plasma will be measured using a validated and sensitive radioimmunoassay that, after extraction, has a plasma limit of detection of 0.2 pmol/L.

Strong evidence suggests that both ANP and BNP regulate energy balance by increasing mitochondrial efficiency and reducing lipogenesis in vitro and in experimental animals in vivo [2]. Our finding that both ANP and BNP in healthy participants at middle age are negatively related to lipid levels and other markers of the metabolic syndrome are consistent with these and other reports [50,51]. This inverse relationship is expected to strengthen over time. Conceivably, by reducing atherosclerotic changes and reducing arterial stiffness, these cardiac hormones ameliorate declines in LV strain and help to maintain high myocardial performance throughout life. Re-examination of cardiac performance after 15 years using speckle echocardiography is expected to reveal differential impacts of ANP and BNP on age-related changes in cardiac contractile function [29]. In this context, it needs to be emphasized that, in health, there is a progressive age-dependent decrease in LV stroke volume and LV volumes and an increase in LV mass along with an increase in LV and arterial elastance in both sexes [52,53]. Aligning with these age-related changes are increases in plasma ANP and BNP. Factors driving this progression are not known, but it is likely that increasing intramural pressure and myocardial inflammation and fibrosis play a major role (reviewed in Sayed et al [54]). Our findings of inverse associations of BNP with LV stiffening at age 28 years and at age 50 years in both sexes (except in older women) are consistent with these changes in chamber structure and function. Plausibly, waning estrogen production in older menopausal women may aggravate myocardial stress [55,56] and initiate an adaptive (compensatory) BNP response in those most at risk by lacking protective gene variants. Notably, a recent cross-sectional study using speckle-tracking echocardiography [29] found that left atrial dysfunction is the earliest sign of age-related cardiac pathology in a population of well people, mean age 65 (range 24-86) years, in which mean BNP level was 2-fold higher than we found in CHALICE. ANP values were not reported. These findings suggest that BNP increases as an adaptive compensatory response to atrial dysfunction [29]—analogous to the loss of the negative association of ANP/BNP with LV elastance we observed in older women (Figure 2). Understanding the impact of the much lower BNP values we identified at 50 years of age with adverse changes in cardiac function 15 years later therefore becomes a pressing need. Based on our findings at age 50 years, we anticipate that the increase in atrial strain will be greater in those with lower ANP and BNP and the percentage change in both peptides at age 65 years will be greater in those with lower levels at baseline (age 50 years). A similar pattern of change in the relationship of LV volumes with ANP and BNP is anticipated. However, it is interesting to note that, in a recent report [18] of the association of a gene variant—rs198389 (increasing BNP)—in preventing LV dysfunction [18] in participants known to be at high risk of developing heart disease, the percentage change in BNP was independent of levels at baseline. Clearly, further studies in healthy participants are needed, preferably when the strong impact of variable age is reduced. As mentioned earlier in this paragraph, a sex difference was noted for older women in whom the negative association of ANP and BNP with LV stiffening is lost. This will be an important finding to re-examine in postmenopausal years, particularly in light of the high incidence of diastolic dysfunction and emergence of heart failure with preserved ejection fraction (HFpEF) in older women [55]. The loss of arterial-LV coupling [57] implied by our findings at 50 years of age in older women—with an associated (adaptive) increase in BNP—suggests that this may portend more severe dysfunction in later years and can be expected to be clarified in this proposal.

### Role of Paracrine CNP

The significance of circulating products of paracrine CNP for cardiometabolic health is less clear than for endocrine-paracrine/BNP [58] largely due to its unique specific (paracrine) actions, rapid degradation at source, and barely detectable levels of bioactive forms (CNP 22 and CNP 53) in adult plasma once growth plates have closed [7]. Nevertheless, there is evidence in experimental animals of important actions in maintaining flow and pressure in the microcirculation [6], counteracting myocardial inflammation and fibrosis [2,23,24], and preserving diastolic function [59,60]. Several studies have suggested these actions come into play after injury (that is, are adaptive) rather than conferring benefit pre-ictus [2,23]. It remains to be seen whether genetic variants increasing CNP/NPR2/cGMP signaling—including loss of function mutations in the natriuretic peptide clearance receptor gene [*NPR3*] [61]—could reduce the incidence of cardiometabolic disease. Other studies of genetic variants—reducing CNP/*NPR3*/Gi P/cAMP activity—reported increased incidence of circulatory disorders and high blood pressure [62]. Links of plasma CNP or NTproCNP with impaired cardiac function or with vascular degenerative disease have been reviewed previously [7] and support the view that the positive association is likely to reflect a compensatory response to loss of structural integrity. Very few, if any, other studies have examined the association of CNP products with measures of cardiac function in community dwellers. However, one large study of 1841 participants reported plasma CNP (using a direct less specific assay without extraction) identified a high-risk cardiovascular phenotype and future risk of myocardial infarction [63]. Consistent with adaptive and protective actions of CNP in humans are our previous findings in healthy volunteers without history of heart disease [64] in whom plasma values of the inactive product of proCNP 1-103 (NTproCNP) and bioactive CNP increased progressively at midlife in both sexes and showed univariate associations with renal function, high blood pressure, and markers of the metabolic syndrome. A key question arising is the likely source(s) of these products in view of the diverse range of tissues expressing *NPPC* [7]. In mature rodents, the vascular endothelium likely makes the major contribution (some 60%) to circulating levels of CNP [23,65]. Positive veno-arterial gradients of CNP across the heart in humans presenting with acute coronary syndrome [66] support contributions from the myocardial, endocardium, or coronary intimal tissues, as does the sustained increase in plasma CNP postictus in participants with acute coronary syndrome [67]. More focused associative studies have shown significant links of CNP products with recognized vascular and metabolic risk factors in both sexes in 2 separate community studies—one at age 28 years and the other at midlife (CHALICE) [15,16]. Importantly in both studies, significant positive associations of CNP products were found with higher arterial and higher LV elastance in both sexes and with lower LV stroke volume and lower LV end diastolic volumes. Likely sources of this increase in CNP are (1) increased vascular endothelial *NPPC* expression in response to shear stress [11] or inflammation [68] and (2) increased *NPPC* expression in LV myocardial tissue inflammation [3,24]. Increase in bioactive CNP rather than both products after cardiac injury [67] likely results from reduced clearance at the source [69,70]. Restudy of CHALICE participants 15 years later, we predict, will show more severe diastolic dysfunction in those with higher CNP levels at age 50 years—and will be greater in women 15 years after menopause [55]. Such findings could establish CNP as an endogenous marker for HFpEF, for which no suitable biomarker is currently available [56]. In this context, it is relevant to note that an increase in plasma NTproCNP was independently linked to adverse outcome in patients with HFpEF [71]. Aligning with CNP’s adaptive role in combatting arteriosclerosis [21,22], we also anticipate carotid artery intimal thickening will correlate with CNP values at 50 years of age and with the proportional increase of CNP above values at baseline.

Taken together, this study has the potential to advance understanding of paracrine CNP’s role in combatting not only disorders of cardiac function but also arterial degenerative changes not otherwise easily addressed in healthy aging humans. Supportive findings of CNP’s independent association can be expected to identify therapeutic targets—for example, novel therapies using CNP agonists (now already approved for use in humans) or new drugs that increase CNP secretion [72] or raise natriuretic peptide levels by extending the half-life in tissues at risk [73].

### Relevance of cGMP

An important additional analyte (cGMP)—not previously measured in CHALICE—could illuminate distinct tissue actions mediated by the membrane-bound guanylyl cyclase receptors NPR1 (activated by ANP and BNP) or NPR2 (activated by CNP). Both particulate receptors are widely expressed within the heart, vasculature, kidney, and brain [2], releasing cGMP into the cell and extracellular fluid after ligand binding [74]. We found that contrived increases in plasma ANP and BNP elicit consistent and proportionate changes in cGMP in plasma or urine, which, importantly, also link with respective changes in organ function [35]. ANP is 3-fold more effective in raising plasma cGMP when compared with BNP [75]. Previous studies have examined associations of plasma cGMP with vascular risk markers, plasma BNP, and cardiac function in community dwellers without heart disease [36,37] and concluded that the positive association of plasma BNP with plasma cGMP resulted from compensatory responses in cardiac secretion to increased myocardial wall stress. Importantly, ANP was not measured. To our knowledge, no study has examined links of each of the natriuretic peptides with cGMP and cardiac function in participants without a history of heart disease. Although contrived increases in circulating CNP levels are much less effective than ANP or BNP in increasing cGMP [75], the importance of cardiac *NPR2* signaling [76] and its diffuse distribution across cardiomyocytes, in contrast to the highly compartmentalized location of *NPR1* in tubular niches in myocardiocytes [59], may facilitate cGMP access to extracellular fluid and plasma. Further, in rodent adult cardiac fibroblasts, cGMP response to CNP far exceeds that from ANP or BNP [77]. Collectively, these findings indicate the need to study putative links of plasma CNP products and cGMP with changes in cardiac structure and function in the course of aging.

### Strengths and Limitations

Compared with other community studies, the number of participants in CHALICE is small. However, the detailed and comprehensive assessment of genetic, metabolic, cardiac, and vascular status together with the complete suite of natriuretic peptide measurements—all undertaken at one site and using in-house validated assays—contribute to the unique features of this longitudinal study. The narrow age band (49 years to 51 years at baseline) eliminates variables related to age, which is a major confounder in most community studies. Although only a single sample for assay of natriuretic peptides is drawn at baseline and follow-up, we found excellent agreement between test and retest on different days in well participants when studied seated, after resting for 15 minutes. Whether associations reflect beneficial genetic effects versus organ adaptive compensatory responses can only be determined by longitudinal studies as planned here. The time span between data collection (around 15 years) encompasses the period after middle age when cardiovascular disorders become overt. For these reasons, our hypothesis—that higher concentrations of ANP/BNP at age 50 years reduces the incidence of impaired cardiovascular health—will be fully tested. Notwithstanding the importance if proven—enabling currently available clinical interventions—the opportunity to link change in function with *NPR1*/cGMP and *NPR2*/cGMP activity can be expected to clarify interactions of endocrine and paracrine components of these important hormones in maintaining cardiovascular health in humans for the first time.

### Conclusions

In view of the high prevalence of cardiovascular disease and its high morbidity and mortality, there is an urgent unmet need for preventative measures including biomarkers of susceptibility that may allow early intervention in those most at risk. The discovery of the role of natriuretic peptides in maintaining a healthy circulation and metabolism opens up new ways of predicting those most at risk. Previous work in community participants free of heart disease showed that low plasma concentrations of ANP and BNP and higher levels of CNP at midlife associate with increases in pathological measures of cardiac and vascular stiffness and impaired metabolic health. This suggests that natriuretic peptide biomarkers will predict adverse outcomes in later years. Proving this, as described here, will lead to earlier detection of those most at risk and may enable novel preventive strategies augmenting natriuretic peptide levels or activity.

## Abbreviations

ANP: atrial natriuretic peptide
BNP: B-type natriuretic peptide
cGMP: cyclic guanosine monophosphate
CHALICE: Canterbury Health, Ageing and Life Course
CNP: C-type natriuretic peptide
CTx: type 1 collagen C-telopeptide
GWAS: genome-wide association study
HFpEF: heart failure with preserved ejection fraction
LV: left ventricle
NPR1: natriuretic peptide receptor 1
NPR2: natriuretic peptide receptor 2
NPR3: natriuretic peptide clearance receptor
NTproANP: aminoterminal atrial natriuretic peptide
NTproBNP: aminoterminal B-type natriuretic peptide
NTproCNP: aminoterminal C-type natriuretic peptide

## Data Availability

The data sets generated after the completion of this study will be available from the corresponding author on reasonable request and dependent on study participant consent.
